# Effect of angiotensin converting enzyme inhibitors and angiotensin II receptor blockers on need for operative intervention for idiopathic adhesive capsulitis

**DOI:** 10.1016/j.jseint.2023.06.008

**Published:** 2023-07-03

**Authors:** Andrew S. Bi, Aidan G. Papalia, Paul V. Romeo, Lauren H. Schoof, Young W. Kwon, Andrew S. Rokito, Joseph D. Zuckerman, Mandeep S. Virk

**Affiliations:** Division of Shoulder and Elbow Surgery, Department of Orthopedic Surgery, NYU Grossman School of Medicine, NYU Langone Orthopedic Hospital, NYU Langone Health, New York, NY, USA

**Keywords:** Adhesive capsulitis, Idiopathic adhesive capsulitis, Frozen shoulder, Angiotensin II receptor blockers, Angiotensin-converting enzyme inhibitors, ACE/ARB

## Abstract

**Background:**

The exact pathogenesis of idiopathic adhesive capsulitis (IAC) is not fully understood, but an inflammatory profibrotic cascade, largely mediated by transforming growth factor-beta 1 (TGF- β1) has been implicated. Angiotensin II receptor blockers (ARBs) and angiotensin-converting enzyme inhibitors (ACE-Is) both decrease the activity of TGF-β1. The aim of this study was to determine the impact of ACE-Is or ARBs use on the need for operative intervention in IAC.

**Methods:**

This was a retrospective cohort study of patients from a single institutional database with IAC, divided into two cohorts, with and without ACE-I and/or ARB use as the primary exposure and a minimum 2-year follow-up. The primary outcome measured was the incidence of operative intervention including manipulation under anesthesia (MUA) and arthroscopic capsular release (ACR). Additional multivariable logistic regression analysis was performed to evaluate associations between ACE-I/ARB use and likelihood of undergoing an operative procedure.

**Results:**

A total of 17,645 patients met inclusion criteria, with 5424 patients in the ACE-I/ARB cohort and 12,221 in the non-ACE-I/ARB cohort. Overall, 422 (2.4%) patients underwent surgical treatment, 378 (2.1%) ACR, and 74 (0.4%) MUA. There was no significant difference between cohorts in the frequency of surgical procedures or time to procedure since diagnosis. There were no significant differences between individual ACE-Is or ARBs, although Losartan was found to have a trend of decreased rate of intervention (31.7% vs. 36.8%, *P* = .209) when compared to patients not on losartan that did not reach statistical significance. Patient factors predictive of undergoing MUA/ACR were diabetes (*P* = .013), obesity (*P* < .001), and male sex (*P* < .001). Increasing patient age reduces the likelihood of undergoing operative intervention, with patients aged 50-70 years (*P* = .022) and age >70 years (*P* < .001) demonstrating reduced odds as compared to patients aged <30 years.

**Conclusion:**

Patients with IAC have an overall low (2.4%) rate of requiring surgical intervention. While the antifibrotic mechanism of ACE inhibitors and ARBs did not significantly affect the rate of requiring surgical intervention, male gender, obesity, younger age, and diabetes, all increased the risk for operative intervention. Losartan, specifically, may have a disease modifying effect on IAC that should be investigated with larger controlled trials.

Idiopathic adhesive capsulitis (IAC) is a self-limiting condition of unknown etiology and is characterized by shoulder pain and decreased active and passive range of motion (ROM).^10^^,^^24^^,^^41^ Its incidence has been reported to be as high as 5% with risk factors such as female sex, diabetes, hypothyroidism, and associated fibrotic disorders such as Dupuytren’s.^8^^,^^13^^,^^19^^,^^22^^,^^26^^,^^36^ Diagnosis is primarily clinical with nonoperative management being the mainstay of treatment.^11^ For severe, unremitting cases, surgical intervention including manipulation under anesthesia (MUA) and arthroscopic capsular release (ACR) may be warranted.^11^

Although the trigger for onset and pathophysiology of IAC is still unknown, it has been theorized the condition follows an inflammatory profibrotic cascade resulting in intense synovitis and capsular thickening. Among numerous proinflammatory mediators, transforming growth factor-beta 1 (TGF- β1) is known to play a key role.^17^^,^^24^^,^^28^^,^^30^^,^^37^^,^^40^^,^^41^ Angiotensin II receptor blockers (ARBs) and angiotensin-converting enzyme inhibitors (ACE-Is), commonly used antihypertensive agents in the renin-angiotensin-aldosterone system, have demonstrated promising results as antifibrotic agents due to their downstream effects of blocking TGF- β1 in nephrology and also more recently in surgical specialties such as plastic surgery and scar formation.^12^^,^^14^^,^^21^^,^^35^^,^^38^ In orthopedics, ACE-Is and ARBs have recently gained attention for their anti-TGF-β1 effects in decreasing fibrosis and promoting cartilage healing.^20^^,^^31^^,^^33^^,^^39^ However, no study to our knowledge has investigated the effects of ACE-Is and ARBs on the natural history of IAC.

The purpose of this study was to compare IAC patients who were prescribed ACE-Is or ARBs with those not on those medications to identify if there were any differences in need for surgical treatment, including MUA or ACR. We hypothesized that the prediagnosis use of ACE-I/ARB medication would be associated with a decreased need for surgical treatment at final follow-up.

## Methods

### Study design

In this retrospective cohort study, patients diagnosed with primary IAC of the shoulder (International Classification of Diseases 10 code M75.0, International Classification Disease 9 code 726.0) between January 2010 and December 2020 were identified from a single institutional database. Inclusion criteria for this study were a diagnosis of IAC, aged 18 years or more at time of diagnosis, and minimum 2-year follow-up. Exclusion criteria consisted of patients who had prior ipsilateral shoulder surgery, those who underwent multiple shoulder procedures during the study period or those who were prescribed ACE-I or ARB after surgery for IAC as described within the single institutional database. Patients were divided into two cohorts using ACE-I and/or ARB use as the primary exposure. The primary outcome measured was rate of operative intervention in form of MUA (Current Procedural Terminology code 23700) and/or ACR (Current Procedural Terminology 29825) following diagnosis of IAC. Covariates of interest included patient age, sex, race, smoking status, body mass index (BMI), and comorbidities (hypertension, diabetes, rheumatoid arthritis, and hypothyroidism).

### Statistical analysis

A priori power analysis was performed with G∗Power Version 3.1.9.7 (Heinrich Heine Universität, Düsseldorf, Germany) using a 2.5% difference in procedure rates as the threshold for clinical significance. With the goal of achieving a minimum of 80% power at an alpha equal to 0.05, it was determined that 714 patients were needed per cohort.

Descriptive statistics were stratified by concomitant ACE-I/ARB use. Chi-squared or Fisher’s exact test was used for analysis of categorical variables and two-sample *t*-test were used for analysis of continuous variables. Categorical variables were reported as frequencies (%) and continuous variables were reported as mean (standard deviation). Multivariable logistic regression analysis was performed to evaluate associations between ACE/ARB use and likelihood of undergoing MUA and/or ACR procedures in patients diagnosed with IAC. All covariates of interest were included in each model to control for confounding. The odds ratios (ORs) and 95% confidence intervals (CIs) were reported. All statistical analysis was performed in Jupyter Notebook Version 6.4.8 (Project Jupyter, New York, NY, USA) using Python programming language. For all analyses, a *P* value of .05 was considered to be statistically significant.

## Results

### Cohort demographics

A total of 17,645 patients (11,715 [66.4%] female, 5930 [33.6%] male) with mean age of 57.8 ± 12.1 years met inclusion criteria and were included in our analysis. There were 422 (2.4%) patients who underwent treatment, with 378 (2.1%) ACR and 74 (0.4%) MUA procedures performed for treatment of IAC. Thirty patients had undergone both MUA and ACR. The ACE-I/ARB cohort consisted of 5424 (30.7%) patients, while the non-ACE-I/ARB cohort consisted of 12,221 (69.3%) patients. The ACE-I/ARB cohort was older (64.0 ± 11.7 vs. 55.1 ± 11.2, *P* < .001), had a higher BMI (29.1 ± 6.2 vs. 26.1 ± 5.6, *P* < .001), and had a higher frequency of comorbidities compared to the non-ACE-I/ARB cohort. A comprehensive comparison of patient demographics is available in Table I.

**Table I:** Comparison of patient demographics.

	ACE-I/ARB use		*P* value
	No, n = 12,221	Yes, n = 5424	
Age, Mean (SD)	55.1 (11.2)	64.0 (11.7)	<.001
Sex, n (%)			
Female	8430 (69.0)	3285 (60.6)	<.001
Male	3791 (31.0)	2139 (39.4)	
BMI, Mean (SD)	26.1 (5.6)	29.1 (6.2)	<.001
Race, n (%)			
African American	1034 (8.7)	764 (14.2)	<.001
Asian	1243 (10.5)	517 (9.6)	
Native American	42 (0.4)	34 (0.6)	
White	6708 (56.6)	2914 (54.3)	
Other Race	1552 (13.1)	744 (13.9)	
Unknown	1281 (10.8)	389 (7.3)	
Smoking Status, n (%)			
Current	594 (4.9)	290 (5.4)	<.001
Former	2595 (21.5)	1591 (29.4)	
Never	7383 (61.1)	3239 (59.9)	
Unknown	1519 (12.6)	291 (5.4)	
Comorbidities, n (%)			
Hypertension	2100 (17.2)	4913 (90.6)	<.001
Diabetes	1297 (10.6)	2643 (48.7)	<.001
Hypothyroidism	1737 (14.2)	1162 (21.4)	<.001
Rheumatoid Arthritis	350 (2.9)	288 (5.3)	<.001
Procedure			
MUA and Arthroscopic release	299 (2.4)	123 (2.3)	.506
MUA	48 (0.4)	26 (0.5)	.487
Arthroscopic release	273 (2.2)	105 (1.9)	.228

*ACE*-*I*, angiotensin-converting enzyme inhibitors; *ARB*, angiotensin II, receptor blockers; *MUA*, manipulation under anesthesia; *SD*, standard deviation.

There were no differences between the frequencies of MUA and/or ACR (123 [2.3%] vs. 299 [2.4%], *P* = .51) between the ACE-I/ARB and non-ACE-I/ARB cohorts. When comparing by individual procedures, there were no significant differences in procedure frequency or time to procedure since diagnosis of IAC for MUA (procedure frequency: 26 [0.5%] vs. 48 [0.4%], *P* = .487; time to procedure in months: 7.4 ± 10.5 vs. 6.1 ± 10.0, *P* = .607) or ACR (procedure frequency: 105 [1.9%] vs. 273 [2.2%], *P* = .228; time to procedure in months: 8.4 ± 15.5 vs. 5.5 ± 12.1, *P* = .138) between ACE-I/ARB and non-ACE-I/ARB cohorts.

### Individual ACE-I/ARB medication use

Of those taking ACE-I/ARB medication, 2487 (45.9%) were on an ACE inhibitor, while the remaining 2904 (53.5%) were on an ARB. On average, the ACE-I/ARB cohort had been on their respective medications for an average of 28.4 ± 29.0 months prior to being diagnosed with IAC. A comprehensive list of medications is available in Table II. The most commonly prescribed medications were Losartan (1989 [36.7%]), Lisinopril (1413 [26.1%]), and Enalapril (601 [11.1%]). Although there were no significant differences between individual ACE-Is and ARBs with respect to their impact on reducing the prevalence of operative intervention, but Losartan demonstrated maximum impact (31.7% vs. 36.8%, *P* = .21) when compared to patients not on losartan.

**Table II:** ACE-I/ARB medication.

	Overall, n = 17,645	MUA and/or arthroscopic release		*P* value
		No, n = 17,223	Yes, n = 422	
ACE-I/ARB Use	5424 (30.7%)	5301 (30.8%)	123 (29.1%)	.506
Class				
ACE-I	2487 (46.1%)	2421 (45.9%)	66 (53.7%)	.109
ARB	2904 (53.9%)	2847 (53.7%)	57 (46.3%)	.112
ACE-I/ARB Name				
Azilsartan Medoxomil	66 (1.2)	66 (1.2)	0 (0.0)	.411
Benazepril	95 (1.8)	91 (1.7)	4 (3.3)	.292
Captopril	23 (0.4)	22 (0.4)	1 (0.8)	.427
Enalapril	601 (11.1)	589 (11.1)	12 (9.8)	.611
Fosinopril	24 (0.4)	24 (0.5)	0 (0.0)	1.00
Irbesartan	228 (4.2)	225 (4.2)	3 (2.4)	.394
Lisinopril	1413 (26.1)	1373 (25.9)	40 (32.5)	.300
Losartan	1989 (36.7)	1950 (36.8)	39 (31.7)	.209
Olmesartan	212 (3.9)	206 (3.9)	6 (4.9)	.846
Quinapril	67 (1.2)	66 (1.2)	1 (0.8)	1.00
Ramipril	268 (4.9)	260 (4.9)	8 (6.5)	.66
Sacubitril	17 (0.3)	17 (0.3)	0 (0.0)	1.00
Telmisartan	89 (1.6)	87 (1.6)	2 (1.6)	1.00
Valsartan	332 (6.1)	325 (6.1)	7 (5.7)	.873

*ACE*-*I*, angiotensin-converting enzyme inhibitors; *ARB*, angiotensin II, receptor blockers; *MUA*, manipulation under anesthesia.

### Multivariable logistic regression for predictors of MUA/ACR

**Figure 1:**
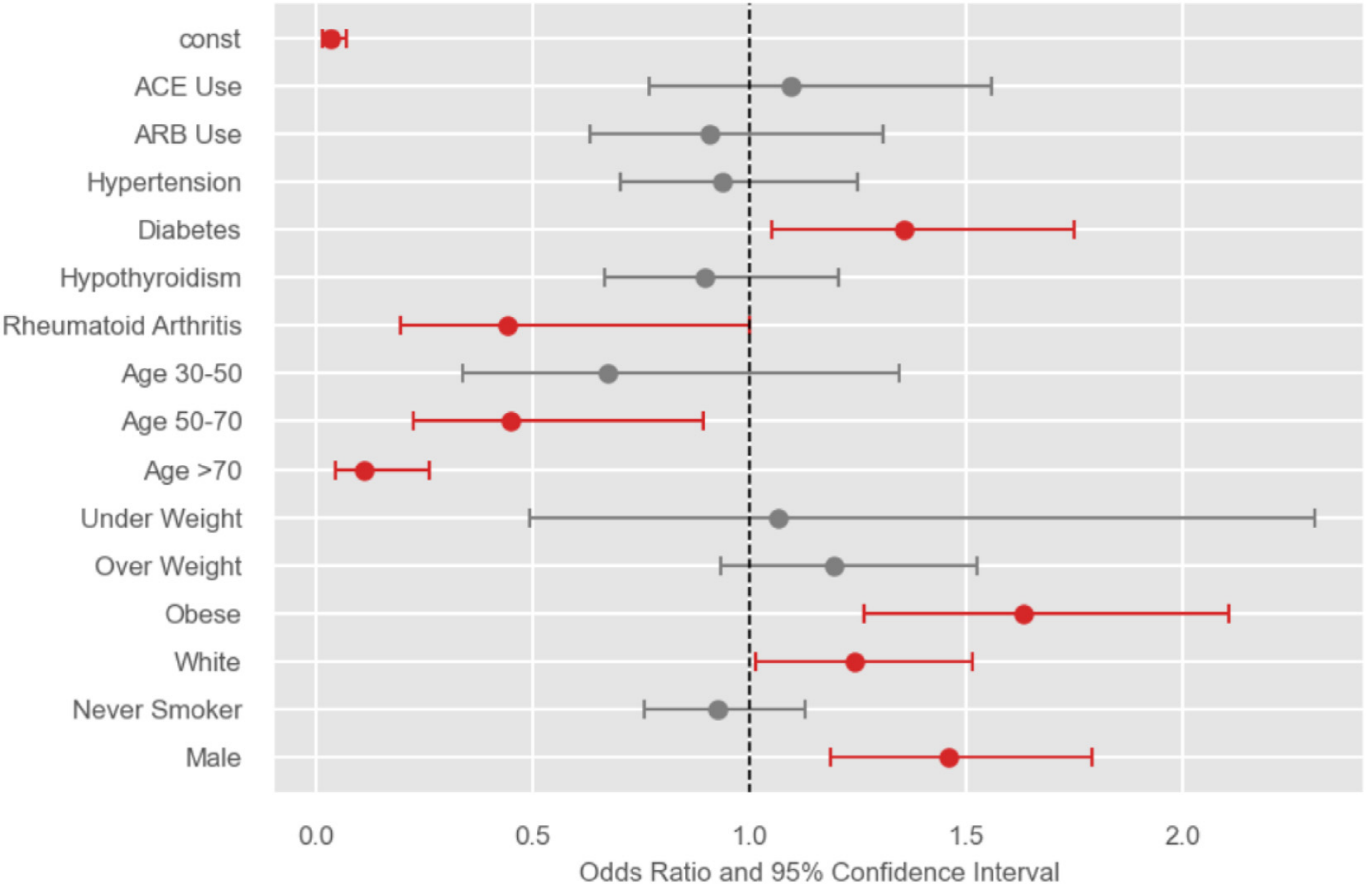
Odds ratios and 95% confidence intervals for factors predictive of undergoing MUA ± capsular adhesion lysis in patients with adhesive capsulitis. *ACE*, angiotensin-converting enzyme inhibitors; *ARB*, angiotensin receptor blocker; *MUA*, manipulation under anesthesia.

A multivariable logistic regression analysis was performed controlling for confounding factors including age, sex, comorbidities, and BMI. Neither ACE-I (OR 1.09, CI 0.76-1.55, *P* = .616) nor ARB (OR 0.90, CI 0.63-1.30, *P* = .609) use were associated with a lower likelihood of undergoing MUA or ACR in patients with IAC. Patient factors predictive of undergoing MUA and/or ACR were diabetes (OR 1.37, CI 1.06-1.77, *P* = .013), obesity (OR 1.62, CI 1.26-2.09, *P* < .001), and male sex (OR 1.46, CI 1.19-1.79, *P* < .001). Increasing patient age appears to reduce the likelihood of undergoing MUA and/or ACR, with patients aged 50-70 years (OR 0.44, CI 0.22-0.89, *P* = .022) and aged >70 years (OR 0.10, CI 0.04-0.25, *P* < .001) demonstrating reduced odds as compared to patients aged <30 years. A comprehensive comparison list of factors and their associated ORs are available in Table III and Figure 1.

**Table III:** Multivariable regression evaluating factors predictive of MUA/arthroscopic release in patients with adhesive capsulitis.

Factor	OR	95% CI	*P* value
ACE-I Use	1.09	0.76-1.55	.616
ARB Use	0.90	0.63-1.30	.609
Hypertension	0.94	0.70-1.25	.678
Diabetes	1.37	1.06-1.77	.013
Hypothyroidism	0.89	0.66-1.20	.465
Rheumatoid Arthritis	0.44	0.19-1.00	.051
Age 30-50 vs. Age <30	0.67	0.33-1.34	.26
Age 50-70 vs. Age <30	0.44	0.22-0.89	.022
Age >70 vs. Age <30	0.10	0.04-0.25	<.001
Under Weight vs. Normal Weight	1.06	0.49-2.30	.87
Overweight vs. Normal Weight	1.19	0.93-1.52	.160
Obese vs. Normal Weight	1.62	1.26-2.09	<.001
White vs. Non-White Race	1.24	1.01-1.51	.034
Never vs. Ever Smoker	0.92	0.75-1.12	.437
Male vs. Female	1.46	1.19-1.79	<.001

*ACE*-*I*, angiotensin-converting enzyme inhibitors; *ARB*, angiotensin II, receptor blockers; *MUA*, manipulation under anesthesia.

## Discussion

The main finding of our study was that ACE-I or ARB use did not have a significant impact on reducing the incidence of surgical intervention (MUA and/or ACR) in patients with IAC. ACE-Is and ARBs are believed to have an antifibrotic mechanism through TGF- β1 modulation in addition to their antihypertensive effect through the renin-angiotensin-aldosterone system;^6^^,^^12^^,^^14^^,^^20^^,^^35^ however, the literature within orthopedics remains in nascent stages. Additionally, we demonstrate that male sex, younger age, obesity, and diabetes were all associated with an increased likelihood of requiring surgical intervention in IAC.

Although the antifibrotic effect of ACE-Is/ARBs has been demonstrated in preclinical models,^20^^,^^29^^,^^33^^,^^39^ the results in orthopedic literature have been mixed. Three recent studies have investigated the effect of ACE-Is/ARBs on the incidence of postoperative arthrofibrosis, ROM, and manipulation in total knee arthroplasty.^2^^,^^18^^,^^25^ None of the three studies demonstrated significant improvement in postoperative ROM, reduction in need for MUA, or decreased revision rate. The most recently published study by Arraut et al evaluated 79 patients undergoing primary total knee arthroplasty who were prescribed losartan at least three months prior to surgery, matched with a control group of patients who were not taking losartan. Postoperative ROM and change in ROM, rates of readmission, manipulation for stiffness, or all-cause revision were not different between groups.^2^ In our study, the lack of difference in rates of operative intervention could be due to multiple reasons. First, the antifibrotic dosage may be different than the antihypertensive dosage for ACE-Is/ARBs. Second, although we did a preliminary power analysis, we may need a larger sample size to demonstrate a clinically significant difference. Finally, while IAC is characterized by fibrosis and contracture of the glenohumeral joint capsule with high expression of TGF-β1, high levels of matrix metalloproteinases, interleukin-1, tumor necrosis factor alpha, and cyclooxygenase (COX1 and COX2) have also been demonstrated to have abnormal expression in IAC tissues and may continue to promote a profibrotic cascade despite the anti-TGF-β1 effects of ACE-Is and ARBs.^17^^,^^28^^,^^30^

Multiple animal models have demonstrated losartan’s ability to inhibit fibrosis, improve skeletal muscle regeneration, and decrease post-traumatic joint capsule stiffness through its antagonism of TGF- β1.^4^^,^^9^^,^^12^^,^^23^ While it did not reach statistical significance, our study demonstrated that patients taking losartan had a trend of decreased rate of surgical MUA and/or arthroscopic release. Losartan is an ARB and prevents direct activation of TGF-β1 as well as the angiotensin II-induced phosphorylation of Smad2 and Smad3, which translocate into the nucleus of the cell and lead to additional increased transcription of TGF-β1, procollagen, and fibronectin.^32^ Losartan specifically has been evaluated in the orthopedic literature for its antifibrotic properties and its mechanistic differences when compared to ACE-I and other ARBs.^7^^,^^15^^,^^41^^,^^42^

In this study, we demonstrate that male sex, younger age, obesity, and diabetes were all associated with an increased likelihood of requiring surgical intervention in IAC. Levine et al reported that patients with more severe initial symptoms, younger age at onset, and reduction in ROM despite 4 months of therapy were more likely to require surgical intervention.^27^ Multiple additional studies have demonstrated an increased rate of surgical intervention in younger patients.^1^^,^^34^ However, it is difficult to ascertain if this is due to more severe unremitting symptoms or if younger patients are less likely to tolerate smaller decreases in ROM and/or surgeons are more aggressive in indicating younger patients for surgical intervention. Some studies have similarly shown an increased rate of surgical intervention among men, as demonstrated in our results, but this is not replicated throughout the literature.^16^^,^^34^ Diabetes is also a significant risk factor for developing adhesive capsulitis and increases the likelihood of requiring surgical management.^3^^,^^16^^,^^31^ Interestingly, BMI has not previously been associated with frozen shoulder, and a recent study investigating the effects of diabetes and BMI on frozen shoulder found no significant difference in surgical requirement or overall outcomes based on BMI more than 30.^5^ Our study demonstrated that obese IAC patients were 1.62 times more likely to undergo surgery.

### Limitations

The retrospective nature of this study introduces the inherent possibility of selection bias and errors in the entered data. Additionally, no ROM outcomes were recorded, preoperatively or postoperatively. This is important as patients with worse presenting ROM have been shown to be more likely to require surgical intervention. Also, while only a small percentage of patients required surgical intervention, it is possible that ACE-Is/ARBs improved ROM without significantly impacting the need for operative intervention. The dosage of ACE-I/ARB medication may also have played a role in the lack of significant findings, as all patients take just their prescribed dose of antihypertensive medication, which was not standardized among patients. However, given there are no clear optimal dosage requirements for the antifibrotic effects of these medications, we felt this lack of dosage recorded would not impact the generalizability of our results significantly. Finally, patient compliance with their ACE-I/ARB medication was also not recorded, which while certainly a limitation, we felt this increased the external validity of the study results, as when investigating such a large cohort of more than 17,000 patients and applying the results to the general population, patients will have varying degrees of compliance in clinical practice.

## Conclusion

Patients with IAC have an overall low (2.4%) rate of requiring surgical intervention. While the antifibrotic mechanism of ACE-Is and ARBs did not significantly affect the rate of requiring surgical intervention, male gender, obesity, younger age, and diabetes all increased the risk for operative intervention. Losartan, specifically, may have a disease modifying effect on IAC that should be investigated with larger controlled trials.

## Disclaimers

Funding: No outside funding or grants were received in support of the completion of this study.

Conflict of Interest: The authors of this paper certify that they have no affiliations with or involvement in any organization or entity with any financial or nonfinancial interests pertinent to the subject matter discussed in this manuscript.

## References

[1] Ando A., Sugaya H., Hagiwara Y., Takahashi N., Watanabe T., Kanazawa K. (2013). Identification of prognostic factors for the nonoperative treatment of stiff shoulder. Int Orthop.

[2] Arraut J., Lygrisse K.A., Singh V., Fiedler B., Schwarzkopf R., Rozell J.C. (2023). The effect of losartan on range of motion and rates of manipulation in total knee arthroplasty: a retrospective matched cohort study. Arch Orthop Trauma Surg.

[3] Balci N., Balci M.K., Tüzüner S. (1999). Shoulder adhesive capsulitis and shoulder range of motion in type II diabetes mellitus: association with diabetic complications. J Diabetes Complications.

[4] Baranowski A., Schlemmer L., Förster K., Slotina E., Mickan T., Truffel S. (2019). Effects of losartan and atorvastatin on the development of early posttraumatic joint stiffness in a rat model. Drug Des Devel Ther.

[5] Barbosa F., Swamy G., Salem H., Creswell T., Espag M., Tambe A. (2019). Chronic adhesive capsulitis (Frozen shoulder): comparative outcomes of treatment in patients with diabetes and obesity. J Clin Orthop Trauma.

[6] Bedair H.S., Karthikeyan T., Quintero A., Li Y., Huard J. (2008). Angiotensin II receptor blockade administered after injury improves muscle regeneration and decreases fibrosis in normal skeletal muscle. Am J Sports Med.

[7] Brown N.J., Agirbasli M., Vaughan D.E. (1999). Comparative effect of angiotensin-converting enzyme inhibition and angiotensin II type 1 receptor antagonism on plasma fibrinolytic balance in humans. Hypertension.

[8] Bunker T.D., Anthony P.P. (1995). The pathology of frozen shoulder. A Dupuytren-like disease. J Bone Joint Surg Br.

[9] Burks T.N., Andres-Mateos E., Marx R., Mejias R., Van Erp C., Simmers J.L. (2011). Losartan restores skeletal muscle remodeling and protects against disuse atrophy in sarcopenia. Sci Transl Med.

[10] Challoumas D., Biddle M., McLean M., Millar N.L. (2020). Comparison of treatments for frozen shoulder: a systematic review and meta-analysis. JAMA Netw Open.

[11] D'Orsi G.M., Via A.G., Frizziero A., Oliva F. (2012). Treatment of adhesive capsulitis: a review. Muscles Ligaments Tendons J.

[12] el-Agroudy A.E., Hassan N.A., Foda M.A., Ismail A.M., el-Sawy E.A., Mousa O. (2003). Effect of angiotensin II receptor blocker on plasma levels of TGF-beta 1 and interstitial fibrosis in hypertensive kidney transplant patients. Am J Nephrol.

[13] Erickson B.J., Shishani Y., Bishop M.E., Romeo A.A., Gobezie R. (2019). Adhesive capsulitis: demographics and predictive factors for success following steroid injections and surgical intervention. Arthrosc Sports Med Rehabil.

[14] Fang Q.Q., Wang X.F., Zhao W.Y., Ding S.L., Shi B.H., Xia Y. (2018). Angiotensin-converting enzyme inhibitor reduces scar formation by inhibiting both canonical and noncanonical TGF-β1 pathways. Sci Rep.

[15] Fogari R., Zoppi A., Preti P., Fogari E., Malamani G., Mugellini A. (2001). Differential effects of ACE-inhibition and angiotensin II antagonism on fibrinolysis and insulin sensitivity in hypertensive postmenopausal women. Am J Hypertens.

[16] Griggs S.M., Ahn A., Green A. (2000). Idiopathic adhesive capsulitis. A prospective functional outcome study of nonoperative treatment. J Bone Joint Surg Am.

[17] Hand G.C., Athanasou N.A., Matthews T., Carr A.J. (2007). The pathology of frozen shoulder. J Bone Joint Surg Br.

[18] Hernandez N.M., Cunningham D.J., Kabirian N., Mont M.A., Jiranek W.A., Bolognesi M.P. (2021). Angiotensin receptor blockers were not associated with decreased arthrofibrosis after total knee arthroplasty. Orthopedics.

[19] Hsu J.E., Anakwenze O.A., Warrender W.J., Abboud J.A. (2011). Current review of adhesive capsulitis. J Shoulder Elbow Surg.

[20] Huard J., Bolia I., Briggs K., Utsunomiya H., Lowe W.R., Philippon M.J. (2018). Potential usefulness of losartan as an antifibrotic agent and adjunct to platelet-rich plasma therapy to improve muscle healing and cartilage repair and prevent adhesion formation. Orthopedics.

[21] Khodaei B., Nasimi M., Nassireslami E., Seyedpour S., Rahmati J., Haddady Abianeh S. (2022). Efficacy of topical losartan in management of mammoplasty and abdominoplasty scars: a randomized, double-blind clinical trial. Aesthetic Plast Surg.

[22] Kingston K., Curry E.J., Galvin J.W., Li X. (2018). Shoulder adhesive capsulitis: epidemiology and predictors of surgery. J Shoulder Elbow Surg.

[23] Kobayashi T., Uehara K., Ota S., Tobita K., Ambrosio F., Cummins J.H. (2013). The timing of administration of a clinically relevant dose of losartan influences the healing process after contusion induced muscle injury. J Appl Physiol (1985).

[24] Kolade O., Ghosh N., Luthringer T.A., Rosenthal Y., Kwon Y.W., Rokito A.S. (2021). Correlation of patient reported outcome measurement information system (PROMIS) with American Shoulder and Elbow Surgeon (ASES), and Constant (CS) scores in idiopathic adhesive capsulitis. J Shoulder Elbow Surg.

[25] Langston J.R., Ramsey D.C., Skoglund K., Schabel K. (2020). Angiotensin II blockade had no effect on range of motion after total knee arthroplasty: a retrospective review. J Orthop Surg Res.

[26] Le H.V., Lee S.J., Nazarian A., Rodriguez E.K. (2017). Adhesive capsulitis of the shoulder: review of pathophysiology and current clinical treatments. Shoulder Elbow.

[27] Levine W.N., Kashyap C.P., Bak S.F., Ahmad C.S., Blaine T.A., Bigliani L.U. (2007). Nonoperative management of idiopathic adhesive capsulitis. J Shoulder Elbow Surg.

[28] Lho Y.M., Ha E., Cho C.H., Song K.S., Min B.W., Bae K.C. (2013). Inflammatory cytokines are overexpressed in the subacromial bursa of frozen shoulder. J Shoulder Elbow Surg.

[29] Logan C.A., Gao X., Utsunomiya H., Scibetta A.C., Talwar M., Ravuri S.K. (2021). The beneficial effect of an intra-articular injection of losartan on microfracture-mediated cartilage repair is dose dependent. Am J Sports Med.

[30] Lubis A.M., Lubis V.K. (2013). Matrix metalloproteinase, tissue inhibitor of metalloproteinase and transforming growth factor-beta 1 in frozen shoulder, and their changes as response to intensive stretching and supervised neglect exercise. J Orthop Sci.

[31] Massoud S.N., Pearse E.O., Levy O., Copeland S.A. (2002). Operative management of the frozen shoulder in patients with diabetes. J Shoulder Elbow Surg.

[32] Mauviel A. (2005). Transforming growth factor-beta: a key mediator of fibrosis. Methods Mol Med.

[33] Premkumar A., Anatone A., Illescas A., Memtsoudis S., Cross M.B., Sculco P.K. (2022). Perioperative use of antifibrotic medications associated with lower rate of manipulation after primary TKA: an analysis of 101,366 patients. J Arthroplasty.

[34] Redler L.H., Dennis E.R. (2019). Treatment of adhesive capsulitis of the shoulder. J Am Acad Orthop Surg.

[35] Robles N.R., Cerezo I., Hernandez-Gallego R. (2014). Renin-angiotensin system blocking drugs. J Cardiovasc Pharmacol Ther.

[36] Rodeo S.A., Hannafin J.A., Tom J., Warren R.F., Wickiewicz T.L. (1997). Immunolocalization of cytokines and their receptors in adhesive capsulitis of the shoulder. J Orthop Res.

[37] Sarasua S.M., Floyd S., Bridges W.C., Pill S.G. (2021). The epidemiology and etiology of adhesive capsulitis in the US Medicare population. BMC Musculoskeletal Disord.

[38] Tan W.Q., Fang Q.Q., Shen X.Z., Giani J.F., Zhao T.V., Shi P. (2018). Angiotensin-converting enzyme inhibitor works as a scar formation inhibitor by down-regulating Smad and TGF-β-activated kinase 1 (TAK1) pathways in mice. Br J Pharmacol.

[39] Utsunomiya H., Gao X., Deng Z., Cheng H., Nakama G., Scibetta A.C. (2020). Biologically regulated marrow stimulation by blocking TGF-β1 with losartan oral administration results in hyaline-like cartilage repair: a rabbit osteochondral defect model. Am J Sports Med.

[40] Zhao H., Kong L., Shen J., Ma Y., Wu Z., Li H. (2021). Tetrandrine inhibits the occurrence and development of frozen shoulder by inhibiting inflammation, angiogenesis, and fibrosis. Biomed Pharmacother.

[41] Zuckerman J.D., Rokito A. (2011). Frozen shoulder: a consensus definition. J Shoulder Elbow Surg.

[42] Zusman R.M. (1999). Are there differences among angiotensin receptor blockers?. Am J Hypertens.

